# Effects of Physical-Cognitive Dual Task Training on Executive Function and Gait Performance in Older Adults: A Randomized Controlled Trial

**DOI:** 10.1155/2016/5812092

**Published:** 2016-12-08

**Authors:** S. Falbo, G. Condello, L. Capranica, R. Forte, C. Pesce

**Affiliations:** Department of Movement, Human and Health Sciences, University of Rome Foro Italico, Rome, Italy

## Abstract

Physical and cognitive training seem to counteract age-related decline in physical and mental function. Recently, the possibility of integrating cognitive demands into physical training has attracted attention. The purpose of this study was to evaluate the effects of twelve weeks of designed physical-cognitive training on executive cognitive function and gait performance in older adults. Thirty-six healthy, active individuals aged 72.30 ± 5.84 years were assigned to two types of physical training with major focus on physical single task (ST) training (*n* = 16) and physical-cognitive dual task (DT) training (*n* = 20), respectively. They were tested before and after the intervention for executive function (inhibition, working memory) through Random Number Generation and for gait (walking with/without negotiating hurdles) under both single and dual task (ST, DT) conditions. Gait performance improved in both groups, while inhibitory performance decreased after exercise training with ST focus but tended to increase after training with physical-cognitive DT focus. Changes in inhibition performance were correlated with changes in DT walking performance with group differences as a function of motor task complexity (with/without hurdling). The study supports the effectiveness of group exercise classes for older individuals to improve gait performance, with physical-cognitive DT training selectively counteracting the age-related decline in a core executive function essential for daily living.

## 1. Introduction

Many activities of daily life involve the simultaneous performance of multiple tasks concurrently challenging motor and cognitive functions. In aging, the ability to perform multiple tasks common in daily living such as walking while engaged in a concurrent mental task (e.g., walking and talking) becomes impaired [1].

Researchers frequently adopt the dual task (DT) technique (i.e., simultaneous performance of two tasks) to explore multitasking ability as well as the effects of different activities or training on executive function. Based on the postulate that the attentional system has a limited pool of resources [2], it is expected that the concomitant performance of different tasks competing for the same resources could be worse with respect to the independent performance of each task. Significant decrements in gait and/or cognitive performance are observed in older adults when cognitive tasks are performed while walking [3, 4]. Such performance decrements are referred to as DT interference and commonly evaluated as the difference between the single and DT performance in relation to the first (DT cost).

One relevant factor underlying this loss seems a disproportional age-related decrease in higher-level cognitive function, the executive [5]. Executive functions are responsible for planning, initiating, sequencing, and monitoring complex goal-directed behaviour as well as controlling complex activities and therefore indispensable for independent daily living and behavioural adaptability [6].

Physical activity and exercise have been demonstrated to induce positive effects on executive function in aging [7], with a large body of research consistently demonstrating the beneficial effect of aerobic exercise on executive function [8]. Recently, research suggests that also forms of exercise different from aerobic, such as strength and coordination [9, 10], may improve cognitive and in particular executive functioning. There is consistent evidence that both physical and cognitive training have the potential to maintain cognitive efficiency in aging [11] and that combining them in successive or simultaneous way amplifies their efficacy [12, 13]. For this reason, physical-cognitive dual tasking is emerging as a novel modality for reaping largest cognitive health benefits [14, 15]. In their review, Wollesen and Voelcker-Rehage [15] highlighted the beneficial effects of locomotor-cognitive DT training for gait and posture performance and for processing speed and executive function. Moreover, the authors indicated the need for an adequate level of task complexity for DT training to be effective and questioned to what extent the locomotor and/or cognitive DT demands contribute to its effectiveness.

Interventional research suggests a causal relationship linking cognitively and physically demanding motor training, as dance, to improvements in executive function [16]. However, evidence of designed physical-cognitive DT training is controversial, as indicated by reviews that highlight the diversity of the employed training tasks and assessment methods [17]. Dual task training largely varies in type and complexity of both locomotor tasks (straight walking, square-stepping) and concurrent mental tasks (memorizing words, reciting poems, and mental computing; 14). Therefore, the state of the art does not allow thorough comparison and optimal forms of integrated physical-cognitive DT training are yet to be identified. Moreover, definitive conclusions are limited by the lack of studies investigating reciprocal DT effects of the gait task on cognitive performance and vice versa of the cognitive task on gait performance [18].

Thus, the aim of the present study was to evaluate whether physical-cognitive DT training specifically challenging executive function is more beneficial for older adults than physical training with lower executive function demands. Thus, we compared the effects of two types of designed physical training similar in functional motor tasks and motor skills. However, these differed in the type and amount of cognitive and specifically executive function demands. Intervention effects on both executive function and gait performances were evaluated in ST and DT conditions in order to obtain information on reciprocal DT effects and to estimate their associations.

## 2. Methods

### 2.1. Participants

Following approval by the Ethics Committee of the University of Rome Sapienza, recruitment was carried out through a senior leisure center. Sample size was calculated based on walking speed as primary end point from an applied perspective, referring to a previous intervention study [19]. Taking into account an 85% power that the study will detect a treatment difference at a two-sided alpha (probability) level of *p* < .05 and an anticipated dropout of 30%, the following calculations indicated a minimum sample size of *n* = 36 (difference between means (*μd* = *μ*1 − *μ*2) = −.130; standard deviation of difference = .220; effect size (*δ* = |*μ*1 − *μ*2|/*σ*) = .591; *n* = 28, *n* + 30% = 36).

**Figure 1:**
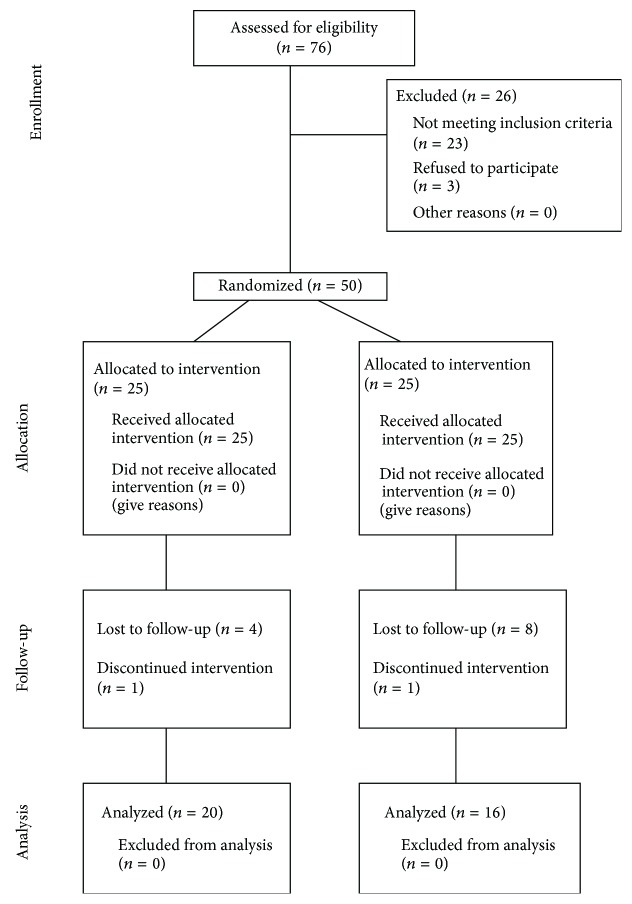
CONSORT flowchart of participants.

Eligibility criteria were age, structured physical activity habits, and medical status that allowed physical training and did not potentially influence study outcomes. Specifically, eligible individuals were men and women aged 65–80 years, participating in structured physical activity no more than twice a week for at least 4 years in a senior leisure center of Rome, without uncontrolled cardiac illness and/or metabolic disease, known history of cerebrovascular disease, or other pathological conditions. The progress through the phases of enrollment, intervention allocation, follow-up, and data analysis is represented in Figure 1. The 50 elderly who agreed to participate signed an informed consent. Two different exercise groups were formed through stratified random sampling, an experimental group exercising with a major focus on physical-cognitive DT training and a control group mainly exercising in ST fashion. Characteristics for stratification were age and general functional ability as judged by the qualified instructor who trained them for the previous 4 years. The 14 participants that dropped out during the program (28%) reported various reasons including development of disease, pains not related to the exercise, anticipation of a scheduled operation, and partner sickness. Therefore, the final number of participants was 36 (means and ± SD: training group with DT focus: 20 of which 2 were men and 18 women, mean age 71.5 ± 6.7 years, weight 65.9 ± 13.1 kg, and height 155.5 ± 8.9 cm; training group with ST focus: 16 of which 2 were men and 14 women, mean age 73.7 ± 4.5 years, weight 65.9 ± 7.9 kg, and height 154.9 ± 7.0 cm).

### 2.2. Testing

Participants were tested twice, before and after the intervention, for executive function and gait performance. Testing was carried out in the facilities of the senior leisure center. Before the testing a trained evaluator gave standardized verbal instructions regarding the tests procedure with a demonstration of all tasks. Participants were then given a practice trial with no recording of performance to ensure familiarization with the tasks. Each test was performed twice and the best trial used for analysis. Validity and reliability of the adopted tests to assess executive function [20, 21] and gait under ST and DT task conditions [22, 23] and of the apparatus to quantify gait parameters [24, 25] have been previously published.

### 2.3. Executive Function

Executive function was tested through the Random Number Generation (RNG) task, a multidimensional test allowing obtaining differentiated indices of core executive functions, inhibition, and working memory updating [26]. Participants were instructed to say a number from 1 to 9 at a constant rate (40 bpm paced by a metronome) such that a requested string of 100 numbers was in an order that was as random as possible. The generated numbers were manually and electronically recorded to elaborate the randomness of the sequence which was measured by different indices.

Six indices reflecting inhibition and working memory updating, three for each function, were obtained (Table 1). For inhibition they were turning point index (TPI), adjacency (ADJ), and runs (Runs). High levels of TPI and low levels of ADJ and runs correspond to a high ability to inhibit, avoiding the production of stereotyped strings and prepotent associates, therefore representing good performance. For working memory the indices were redundancy (RED), coupon (Coupon), and mean repetition gap (MeanRG). High levels of MeanRG and low levels of R and coupon correspond to a high ability to update working memory and employ equality of responses by alternating numbers therefore representing a good performance. For more details on meaning and computation of the above indices see [27].

Two summary indices were calculated for each function. Before averaging, data were *z* standardized and, since high TPI and RGMean values reflect high ability, while high ADJ, runs, redundancy, and coupon values reflect low ability, the latter values were reversed.

### 2.4. Gait

Gait performance was assessed through gait analysis using a photocell system (Optojump Next, Microgate, Bolzano, Italy [24]). The Optojump system used in this study consists of 10 transmitting and 10 receiving optical bars placed parallel to each other at a distance of 2 m, for a total length of 10 m, each containing 96 LEDs. The LEDs on the transmitting bar communicate continuously with those on the receiving bar. The system detects any interruptions in communication between the bars and calculates their duration, in order to measure parameters connected to gait performance.

Participants were asked to walk at their habitual speed, wearing their own footwear between the bars on a rectangular path of 10 × 2 m, for 2.5 min. Gait parameters were recorded only when passing across the bars. To exclude acceleration and deceleration phases from the analysis of the gait parameters the first and the last bars of the Optojump (the first and the last meters) were not considered: acceleration and deceleration phases. The walking task was performed in two conditions: flat and negotiating two hurdles of different heights (6 and 30 cm) added both ways, at 4 and 6 m, respectively.

Quantitative gait parameters were provided by the system (Optojump Next, Microgate, Bolzano, Italy; software version 1.9.7.0). We selected gait speed, stride length, and time for their sensitivity to detect or predict age-related decline in executive function [4] and gait instability in dual tasking [28]. Gait speed was determined by dividing the total distance walked by the duration of the walk time (m/s); stride length (m) is the the distance between heel points of two consecutive footfalls of the same foot. It was obtained normalizing by height; stride time was the duration of the gait cycle that is the time from initial contact of one foot to subsequent contact of same foot (s). For all three selected parameters we computed not only average values, but also coefficients of variation (CV, i.e., standard deviation of the measurement divided by its mean value in % [29]), because variability is considered a better indicator of the degree of dynamic self-organization of the motor system than the central tendency [30].

### 2.5. Task Conditions and Dual Task Interference

**Figure 2:**
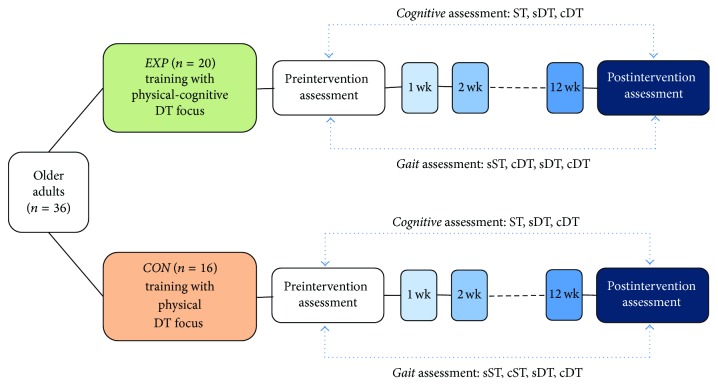
Method schematic. Cognitive assessment (RNG) conditions: (1) ST = single task (i.e., RNG only), (2) sDT = simple gait-cognitive dual task (i.e., flat walking + RNG), and (3) cDT = complex dual task (i.e., walking while negotiating hurdles + RNG). Gait assessment conditions: (1) sST = simple single task (i.e., flat walking), (2) cST = complex single task (i.e., walking while negotiating hurdles), and (3) sDT = simple dual task (i.e., flat walking + RNG) and cDT = complex dual task (i.e., walking while negotiating hurdles + RNG). EXP = experimental group; CON = control group.

The above walking and executive function tests were performed by participants as single task (ST) and combined in DT. Both ST and DT were simple or complex depending on the gait task demands (flat walking versus negotiating hurdles, resp.) as outlined in Figure 2. These experimental conditions were performed in counterbalanced order to avoid practice effects.

To analyze dual task interference, we calculated relative dual task effects (DTE) on both gait and cognitive performance as follows [31]:

(1) $$\begin{array}{c} DTE=\left[\;\frac{\left(dual\;\;task-single\;\;task\right)}{single\;\;task}\;\right]\times 100\%. \end{array}$$

In the case of average stride time and CVs of all gait variables, for which the higher the value, the worse the performance, DTEs were calculated altering the formula as follows [31]:

(2) $$\begin{array}{c} DTE=\left[\;\frac{-\left(dual\;\;task-single\;\;task\right)}{single\;\;task}\;\right]\times 100\%. \end{array}$$

In this way, for all variables, negative DTE values indicate deteriorated performance in DT (i.e., dual task cost), whereas positive values represent an improvement in DT with respect to ST (i.e., dual task benefit).

### 2.6. Exercise Training

Participants of both groups, led by a qualified instructor, exercised with music for 1 hour, twice weekly, for 12 weeks in group-based exercise classes of 25 participants each. Attendance to the intervention program was 85% in both groups. Each training session comprised a 10-minute warm-up made of walking at different speeds, light running, and moving different body segments: arms, wrists, fingers, shoulders, legs, and ankles. This part leads to a 30-minute period of coordination training (e.g., walking with arms circles), balance (e.g., maintaining a monopodalic stance with and without swinging the free leg), strengthening (e.g., squatting while extending an elastic band with arms), agility (e.g., walking through an agility ladder at different speed), followed by 20 minutes of stretching, strengthening and relaxation with exercises alternating contraction and decontraction of muscles coupled with breathing, and slow rotations of hands, head, and ankles performed lying on the floor. Our exercise training types did not have the characteristics of duration, intensity, and overload of aerobic or progressive resistance training. Instead, our general exercise mode fits the description of coordination training by Voelcker-Rehage and Niemann [10], which involves continuous perceptual-motor adaptations to different task requirements. In fact, in the present study, participants of both groups were required to walk in ST (only walking) or DT combination with other bodily movement (e.g., circling, swinging arms) and/or handling small tools (e.g., throwing and catching soft balls), changing walking patterns (e.g., on toes or heels) direction (forward versus backward), and/or speed (slow versus fast) in response to stimuli.

For the experimental group, the physical training tasks were associated with concomitant cognitive tasks specifically relying on executive function. The goal was to engage the three core executive functions: inhibition (the ability to inhibit automated responses), working memory (the ability to hold, process, and manipulate information in mind) and shifting (the ability to change stimulus-response associations for performing an ongoing task). Thus we created gross-motor training conditions that mirrored the cognitive demands of typical frontal tasks created in neuropsychology to tap the activity of the main neural substrate of executive function, the frontal cortex [19]. For example, during the performance of physical tasks, several features of equipment (i.e., colour and/or size of obstacles) were associated with different motor requirements and participants were required to switch randomly between stimulus-response sets. These are characteristics of set-shifting derived from the Wisconsin Sorting Card Test [32].

Moreover, according to evidence that motor and cognitive benefits at old age may be obtained by means of DT training with a certain level of task specificity and rising difficulty [15], further DT experiences with such characteristics were embedded in the exercise training of the experimental group. Task specificity was ensured using functional mobility tasks common in everyday life, as walking on uneven or narrow surface, or carrying objects, walking while talking, or picking up objects off the floor. To ensure that the effects of learning those specific tasks could be disentangled from the pursued improvements in underlying cognitive and motor functions [17], we employed a variety of functional mobility training tasks that did not include those used for testing.

### 2.7. Statistical Analysis

Data were analyzed using the Statistical Package for the Social Science, version 21.0 (SPSS Inc., Chicago Illinois). The level of statistical significance was set at *p* < .05 for all computations. All data were checked for normality of distribution.

One-way multivariate analyses of variance (MANOVAs) and subsequent ANOVAs were run on executive function and gate variables, with group (experimental versus control) as factor to verify whether the two groups were comparable at the beginning of the intervention. To answer the study question on intervention effects, gait and cognitive performance were analyzed as primary outcomes measures and reciprocal DTEs as secondary outcome measures.

Average values and CVs of gait speed, stride length, and stride time were separately submitted to 2 × 2 × 2 × 2 mixed model MANOVAs, with group as the between-participants factor and time (pre versus post), motor complexity (flat versus hurdling), and cognitive complexity (ST without RNG versus DT with RNG) as within-participants factors.

Indices of inhibition and working memory updating and DTEs on cognitive efficiency were submitted to 2 × 2 × 3 mixed model MANOVA, with group and time and task complexity (ST, sDT, and cDT) as factors. In case of significant interactions involving the factors time and group, correlation analyses were performed between pre-post difference values (Δ) of cognitive and motor variables to check for associations between cognitive and gait performance gains.

Regarding the DTEs on cognitive and gait performance, they were also submitted to MANOVAs followed by ANOVAs. The analysis model was a 2 × 2 × 2 mixed model with group (experimental versus control) as between-participants factor and time (pre versus post) and motor complexity (flat versus hurdling) as within-participants factors.

Effects sizes were calculated as partial eta squared (*η*_*p*_^2^) for ANOVA results. Post hoc planned pairwise comparisons through *t*-tests with Bonferroni correction for multiple comparisons were performed in case of significant interactions or main effects for factors with more than two levels.

## 3. Results

There were no significant group differences in executive function (Table 2) or gait performance (Table 3) at preintervention testing time.

**Figure 3:**
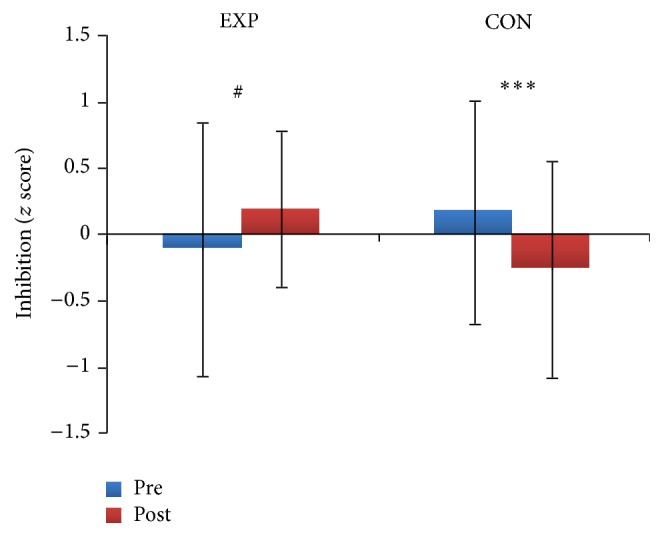
Training effects on inhibitory performance. Significant decrement (*∗∗∗* = *p* < .001) was seen in the control group (CON), while a marginally significant increment (# = *p* < .041) was seen in the experimental group (EXP).

Regarding the effects of training on executive function, there was a significant time × group interaction (*Wilks λ* = .73, *F*_(3,32)_ = 12.4, *p* = .001, and *η*_*p*_^2^ = .27). ANOVA results revealed the presence of this interactive effect on the summary inhibition index (*F*_(1,34)_ = 4.5). Post hoc analysis (adjusted *p* for 2 comparisons = .025) showed a decrement of inhibitory performance after the intervention in the control group (*p* < .001), but a marginally significant increment in the experimental group (*p* = .041; Figure 3).

**Figure 4:**
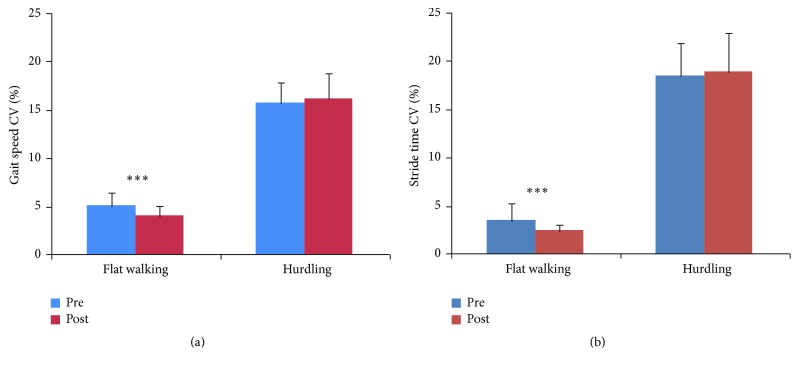
Coefficients of variations of gait speed (a) and stride time (b) following the intervention in simple (flat) and complex (hurdling) walking task conditions. *∗∗∗* = *p* < 0.001.

Regarding the effects of training on gait performance, there was a significant time × motor complexity interaction (*Wilks λ* = .69, *F*_(3,32)_ = 4.17, *p* = .008, and *η*_*p*_^2^ = .31). ANOVA results revealed significant effects on the variability of temporal gait parameters only (gait speed: *F*_(1,34)_ = 10.05, *p* = .003, and *η*_*p*_^2^ = .23 and stride time CV: *F*_(1,34)_ = 13.63, *p* < .001, and *η*_*p*_^2^ = .29). Post hoc analysis (adjusted *p* for 2 comparisons = .025) showed a gain in walking speed variability (*p* < .001; Figure 4(a)) and a decrement of stride time variability (*p* < .001; Figure 4(b)) after the intervention only in the flat walking condition, but not in walking while negotiating hurdles.

No main effects of time or significant time × group interactions emerged for any of the DTE variables.

**Figure 5:**
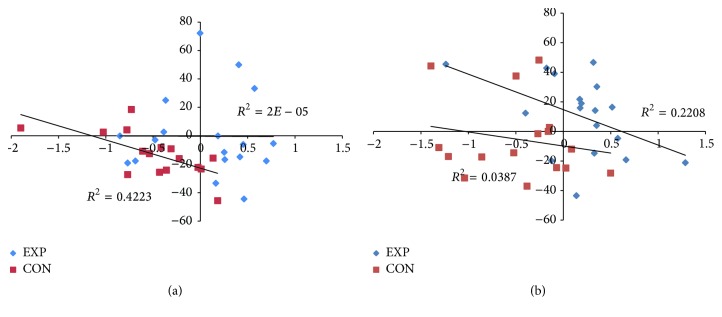
Correlations between pre-post changes in inhibition (horizontal axis) and coefficients of variations of stride length (vertical axis) in simple (panel (a)) and complex (panel (b)) gait-cognitive task conditions. Regression lines represent significant correlations found for the control group (CON) in the simple condition, but for the experimental group (EXP) in the complex condition.

According to the study question, it was finally verified whether the differential pre-post change in inhibition in the two intervention groups was associated with changes in gait performance, as reflected in the average values and CVs of all gate parameters. Significant correlations (Pearson's *r*) of Δ inhibition emerged with Δ values of stride length CV only (Table 4). The correlation between Δ inhibition and Δ stride length CV in ST, considered a general estimate of association between changes in inhibition and gait performance, was not significant. Instead, in line with the focus of the intervention program on DT training, we found significant correlations between Δ inhibition in DT and Δ gait variables in the corresponding DT conditions. Since visual inspection of regression slopes suggested the presence of outliers that might affect those correlations, a multiple regression analysis was conducted with the Δ scores of interest (Δ inhibition and Δ stride length CV in ST and DT conditions) as predictors and an unrelated variable (BMI) as dependent. Two cases (of the experimental group) were detected as outliers and excluded based on Mahalanobis' distance. Significant correlations are represented in Figure 5 separately for the experimental and the control group. In the simple gait-cognitive task condition (sDT), Δ inhibition and Δ stride length CV were significantly correlated in the control group only (Figure 5(a)). Instead in the complex gait-cognitive condition (cDT), they were significantly correlated in the experimental group only (Figure 5(b)).

## 4. Discussion

This work investigated the effects of a physical-cognitive DT training specifically tailored to challenge executive function by movement on cognitive and gait performance in older individuals. How to combine or integrate motor and cognitive demands in physical training represents a recently growing line of research across the lifespan in exercise science [33]. In aging research, specific forms of DT training with cognitive-motor interference and multitask balance training have been developed and demonstrated to benefit both gait and cognitive performance [13, 15, 34]. In the present study, physical training was rendered cognitively challenging by integrating executive function demands in DT fashion and its effects were compared to those of mainly physical training in ST fashion. Both types of training elicited improvements in gait performance, confirming the efficacy of well-designed exercises for older adults [35], but only the physical-cognitive DT intervention contributed to counteracting the age-related decline of inhibitory efficiency. Moreover, changes in inhibitory efficiency during DT walking were paralleled by corresponding changes in gait performance. This may have relevant positive implications to counteract the decreasing ability of older adults to cope with more than one task at a time, as it is common in everyday life.

In contrast to our expectation, gains in gait performance emerged independently of the presence/absence of a concomitant cognitive task, as DT costs seemed unaffected by training. Moreover, the beneficial effect of training was observed when the motor conditions of the testing task were relatively easy, that is, when the gait task was simply flat walking at self-paced habitual walking speed (Figures 4(a) and 4(b) left), but absent when the walking task involved negotiating hurdles (Figures 4(a) and 4(b) right). Presumably to impact the ability to perform more complex walking movements, a longer training duration [16] and/or the inclusion of strength-enhancing progressive resistance training exercises [36] is needed. An alternative interpretation of the improvement in gait performance observed in both intervention groups is learning/habituation due to task repetition. Nevertheless, a learning effect should affect gait parameters more broadly. Instead, the observed improvements selectively regarded the temporal training intervention.

Stride time variability is an indicator in inverse relationship with gait performance: the higher the variability, the lower the performance [37]. In fact, it seems related to motor control of the rhythmic gait patterning, which is well recognized as a crucial aspect of efficient locomotion [38]. More generally, high variability in the performance of a motor coordination task is considered an indicator of higher allocation of attention and cognitive control [39]. Thus, the reduction of speed and stride time variability after training suggests that older adults became better able to maintain a constant gait rhythm with little involvement of cognitive control. This youth-like amelioration is relevant when considering that untrained older adults lose movement automation, require more cognitive resources for planning and controlling walking movements [40], and generally overengage prefrontal areas during motor planning to compensate age-related decline [41].

The two types of exercise training differentially impacted the efficiency of inhibitory functions (Figure 3). The inhibitory deficit hypothesis of aging suggests that many age-related cognitive and social deficits depend on poor inhibitory control [42, 43]. Although this hypothesis and the underlying construct validity of inhibition in older adults are still issues of debate [44, 45], our results suggest that at least the ability to inhibit mental routines worsens in older adults, unless its deterioration is actively counteracted by designed, physical-cognitive DT training. This finding extends and further specifies the notion that physical activity training has the potential to induce cognitive plasticity in older adulthood [11], thus preserving the efficiency of supervisory brain systems in which inhibition is involved [46].

Following the suggestion to investigate reciprocal DT effects on both gait and cognitive task [18], we further verified whether the differential cognitive outcomes of the two training types were associated with corresponding changes in gait performance, specifically during gait-cognitive DT. We found that postintervention changes in inhibitory efficiency while walking were paralleled by changes in gait performance, as reflected in the variability of spatial gait characteristics (Figure 5). Age-related negative changes in gate pace have been associated with decline in executive functions and incremental changes in gait variability with a greater risk of developing cognitive impairment [4]. Conversely, our results suggest that the same association can be positive: training executive functions within physical training elicits inhibitory function gains associated with decremental changes in gait variability.

Interestingly, we found group differences in the associations between changes in inhibition and gait. Older individuals who practiced exercise mainly focused on physical ST training showed deteriorated inhibition paralleled by a decremental change in gait performance under simple DT conditions (Figure 5(a)). The deterioration of inhibitory function is indicated as detrimental to many functional and social activities of daily life. This is the case when, for instance, in the urban environment pedestrians must react to sudden changes by quickly interrupting an ongoing action and selecting a new, appropriate one, or when routine thoughts must be stopped to effectively interact in dialogue with others. In contrast in the case of older participants to the exercise training focused on physical-cognitive DT, this association was found under more complex DT conditions (Figure 5(b)). Speculatively, the maintenance/amelioration of inhibitory efficiency could limit dysfunctional comovements and coactivation particularly impinging on complex movement actions in older individuals. The coactivation of agonist and antagonist muscle in locomotor coordination which typically emerges in aging is responsible for increased metabolic cost and therefore decreased efficiency of walking [47, 48] which limits the performance of all those daily activities requiring active commuting. This finding adds to the notion that designed physical training for older adults, as compared to less focused physical activity programs, has the potential to strengthen the association between physical and cognitive performance [49]. Nevertheless, no association emerged between DT costs/benefits calculated as DTE relative to the performance of the same gait and cognitive tasks in isolation (ST). The high interindividual variability may be responsible for this lack of alignment of cognitive and motor performance changes expressed in relative terms.

The presence of intervention effects on inhibitory function but not on working memory should be discussed referring to the diversity of outcomes and mediators of different physical training types on executive functions in older adulthood. While aerobic exercise is the most acknowledged form of exercise to reap cognitive benefits through cardiovascular and neurotrophic mechanisms inducing changes in brain health and activation, more recently, other types of exercise besides the cardiovascular one have attracted the attention of exercise and cognition researchers [10]. Muscular resistance training of yearly [45] or even monthly duration [50] seems to improve inhibition by enhancing functional plasticity of the cortex associated with inhibition processes [51]. This was observed, for example, with women aged 65 to 75 years by means of high intensity resistance training, even only once a week, of major muscle groups (arms flexions and extensions, seated row, upper limbs pull downs, leg press, legs flexions, and raises on ball of the foot), paralleled by specific strategies to promote participants' engagement [52].

Our findings of intervention effects on inhibition after a three-month intervention add to the evidence that training on different time scales may be efficacious, depending on the quantitative and qualitative characteristics of the designed exercise tasks [14]. Coordination training, characterized by qualitatively variable movement combinations, seems to improve executive functions as cardiovascular exercise, but through different neural mediating mechanisms [53]. Our exercise training joining in DT fashion qualitatively variable movement combinations with specific executive function demands had cognitive outcomes in line with those of studies showing selective benefits for inhibition [54] and no differences for working memory between physical training types that challenged cognition to a greater or lesser degree (i.e., virtual reality videogame dancing versus treadmill walking complemented with strength and balance exercises [16]).

The study has limitations that must be addressed. The small sample size was not powered formally for all variables, but only for a walking variable, as the present work was intended to produce data necessary to adequately power a full scale study. Since, in the present study, no intervention effects were found on any reciprocal DT effect variable, such measures should be prioritized for power calculation in a following full scale study, with power estimates for main variables of both gait and cognitive performance. The choice of individuals already involved in structured physical activity training was made to test whether older individuals may profit from DT training as an added value beyond the benefits of being physically active. Recruitment from the same community center was performed to have physically active older participants avoiding transportation problems but involved the risk of cross-contamination. No measurement was performed to ascertain maintenance of effects after exercise cessation. Methodological differences and particularly the difficulty in operationalizing the breadth of the exercise quality construct in exercise and cognition research [33], categorizing levels of task complexity in group-based training, limit the comparability with previous studies. Particularly the effects of physical-cognitive DT training can strongly vary across studies depending on motor and cognitive task complexity, specificity, and prioritization [15]. The absence of intervention outcomes on DTE in the present study might be due to the relatively short duration and low volume of training, especially considering the low stepwise progression of task complexity due to the time older adults needed to familiarize with variation of the training tasks proposed to mirror the characteristics of executive function tasks.

In conclusion with the present intervention, we aimed to go beyond exclusively physical training, adding physical-cognitive DT demands specifically designed to challenge executive functions. The results support the usefulness of exercise training to enhance gait performance in general, as reflected by the temporal parameters of gait performance and of designed physical-cognitive DT embedded into exercise training to benefit inhibitory efficiency. The training-induced improvement in inhibition, in turn, seems linked to an ameliorated control over the spatial characteristics of gait. From a holistic approach to health promotion, efficient executive function per se is not sufficient for older individuals to perceive health and quality of life, but it must be coupled with tangible experience of ability to walk under dual task conditions that mirror everyday life demands [55]. Our operationalization of physical-cognitive DT training targeted to improve executive function and gait performance jointly seems a suitable means of pursuing such holistic goal. Future research should focus on how well-designed training programs for older adults may positively impinge on the reciprocal influence between cognitive and gait performance that is relevant, if not even crucial, in many everyday life situations.

## Figures and Tables

**Table 1:** Description of the executive function indices obtained from the Random Number Generation test.

Inhibition	
Turning point index (TPI)	Measure of the similarity between the real frequencies of turning points, marking a change between ascending and descending series of numbers and their theoretical frequency in random responses
Adjacency	Measure of the relative frequency of pairs of adjacent ascending or descending numbers and reflecting the habitual tendency to count forward or backward
Runs	Index of variability of the number of digits in successive ascending or descending runs

Working memory	

Redundancy	Measure reflecting the unbalance of response alternative frequencies that derives from a more frequent usage of given numbers as compared to others in a sequence of generated numbers based on the theoretical frequencies of each digit
Coupon	Measure of the mean number of responses given before all the alternative responses are used
Mean repetition gap	Average quantity of digits between successive occurrences of the same number calculated for all digits throughout the whole sequence

**Table 2:** Means and standard deviations of executive function variables obtained through RNG task before and after intervention in all testing conditions: single task (ST) and simple dual task (sDT) while flat walking and complex dual task (cDT) while walking with hurdles.

	Cognitive task (ST)	Simple gait-cognitive task (sDT)	Complex gait-cognitive task (cDT)
Inhibition (summary score)			
Pre	0.00 ± 0.89	0.02 ± 0.88	0.01 ± 0.85
Post	−0.01 ± 0.93	−0.02 ± 0.90	−0.01 ± 0.90
Inhibition (DTE)			
Pre	/	0.02 ± 0.56	0.00 ± 0.61
Post	/	0.00 ± 0.61	0.01 ± 0.60
Working memory (summary score)			
Pre	0.01 ± 0.97	0.02 ± 0.90	−0.01 ± 0.84
Post	0.05 ± 0.53	0.03 ± 0.83	0.00 ± 0.43
Working memory (DTE)			
Pre		0.03 ± 1.03	
Post			

DTE = [(dual task − single task)/single task] × 100%.

**Table 3:** Means and standard deviations of gait performance variables before and after intervention in all testing conditions: simple task (ST) flat walking, complex single task (cST) walking with hurdles, simple dual task (sDT) flat walking with RNG, and complex dual task (cDT) walking with hurdles and RNG.

	Simple gait (sST)	Complex gait (cST)	Simple gait-cognitive task (sDT)	Complex gait-cognitive task (cDT)
Speed average				
Pre	1.24 ± 0.14	1.06 ± 0.12	1.11 ± 0.20	0.99 ± 0.17
Post	1.26 ± 0.16	1.24 ± 0.14	1.16 ± 0.19	1.00 ± 0.15
Speed CV				
Pre	4.48 ± 2.38	16.07 ± 3.46	5.55 ± 3.50	15.45 ± 2.98
Post	3.73 ± 1.28	16.35 ± 3.65	4.30 ± 1.60	15.83 ± 3.16
Speed CV DTE				
Pre			41.31 ± 104.40	−2.09 ± 15.75
Post			28.58 ± 68.93	−1.25 ± 16.52
Stride length average				
Pre	0.80 ± 0.06	0.79 ± 0.07	0.78 ± 0.06	0.78 ± 0.07
Post	0.80 ± 0.08	0.79 ± 0.08	0.78 ± 0.08	0.79 ± 0.08
Stride length CV				
Pre	3.71 ± 1.48	8.84 ± 2.12	3.69 ± 1.63	8.61 ± 2.12
Post	3.14 ± 0.96	8.63 ± 1.59	3.13 ± 1.16	8.50 ± 2.21
Stride length CV DTE				
Pre			5.35 ± 45.32	−0.15 ± 24.02
Post			5.52 ± 43.37	−0.86 ± 21.35
Stride time average				
Pre	1.0 ± 0.08	1.20 ± 0.08	1.13 ± 0.26	1.29 ± 0.16
Post	1.00 ± 0.08	1.20 ± 0.09	1.06 ± 0.13	1.28 ± 0.12
Stride time CV				
Pre	3.08 ± 2.24	18.68 ± 3.81	3.62 ± 2.75	18.31 ± 3.45
Post	2.06 ± 0.56	18.99 ± 4.22	2.74 ± 1.34	18.86 ± 4.16
Stride time CV DTE				
Pre			48.08 ± 115.49	−0.35 ± 15.80
Post			45.18 ± 82.01	0.55 ± 14.15

**Table 4:** Pearson's correlation between pre-to-post changes (Δ) in cognitive efficiency (inhibition performance: the higher, the better) and gait performance (stride length variability: the lower, the better) under single or dual task conditions with simple or complex gait demands (without or with hurdling). EXP = experimental group with DT focus; CON = control group with ST focus.

Δ inhibition	Δ stride length CV			
	Simple gait single task (sST)	Complex gait single task (cST)	Simple gait-cognitive dual task (sDT)	Complex gait-cognitive dual task (cDT)
Cognitive single task (ST)	EXP: *r* = − .033 (*p* = .896)	EXP: *r* = − .080 (*p* = .752)		
	CON: *r* = − .247 (*p* = .356)	CON: *r* = .087 (*p* = .748)		
Simple gait-cognitive dual task (sDT)			EXP: *r* = −.004 (*p* = .986)	
			CON: **r** = −.650^**∗∗**^ (*p* = .006)	
Complex gait-cognitive dual task (cDT)				EXP: **r** = −.470^**∗**^ (*p* = .049)
				CON: *r* = −.197 (*p* = .465)

*∗* = *p* < 0.05; *∗∗* = *p* < 0.01.
